# Reversible Electronic Solid–Gel Switching of a Conjugated Polymer

**DOI:** 10.1002/advs.201901144

**Published:** 2019-10-28

**Authors:** Johannes Gladisch, Eleni Stavrinidou, Sarbani Ghosh, Alexander Giovannitti, Maximilian Moser, Igor Zozoulenko, Iain McCulloch, Magnus Berggren

**Affiliations:** ^1^ Laboratory of Organic Electronics Department of Science and Technology Linköping University SE‐60174 Norrköping Sweden; ^2^ Wallenberg Wood Science Center Department of Science and Technology Linköping University SE‐60174 Norrköping Sweden; ^3^ Department of Chemistry and Centre for Plastic Electronics Imperial College London London SW7 2AZ UK; ^4^ Physical Sciences and Engineering Division KAUST Solar Center (KSC) King Abdullah University of Science and Technology (KAUST) KSC Thuwal 23955–6900 Saudi Arabia

**Keywords:** conjugated polymers, electroactive materials, hydrogels, volume change

## Abstract

Conjugated polymers exhibit electrically driven volume changes when included in electrochemical devices via the exchange of ions and solvent. So far, this volumetric change is limited to 40% and 100% for reversible and irreversible systems, respectively, thus restricting potential applications of this technology. A conjugated polymer that reversibly expands by about 300% upon addressing, relative to its previous contracted state, while the first irreversible actuation can achieve values ranging from 1000–10 000%, depending on the voltage applied is reported. From experimental and theoretical studies, it is found that this large and reversible volumetric switching is due to reorganization of the polymer during swelling as it transforms between a solid‐state phase and a gel, while maintaining percolation for conductivity. The polymer is utilized as an electroactive cladding to reduce the void sizes of a porous carbon filter electrode by 85%.

## Graphical abstract

Conjugated polymers exhibit electrically driven volume changes when electrochemically switched. Here, a thiophene‐based polymer reversibly expands by 300% upon addressing, relative to its previous contracted state, while the first irreversible actuation can achieve values ranging from 1000–10 000%, depending on the applied voltage. Molecular dynamics reveal that the polymer transforms between a solid state to gelled state.

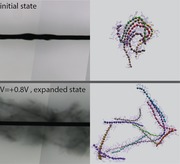

Materials that change in volume upon exposure to external stimuli have been included as the active layer in actuators, robotics, (micro‐)electromechanical systems, biomedical, and fluidic devices.^1^, ^2^ In this context, conjugated polymer films have been explored, since they can exhibit reversible dimensional changes upon electrochemical doping, a process which requires ions, provided from an electrolyte, to enter the film in order to counterbalance accumulated charges along the polymer backbone. The dimensional changes occur thanks to the inclusion of ions and water molecules, as well as due to conformational changes of the polymer chains.^3^, ^4^ Polypyrrole (PPy) has been the most widely used and studied conjugated polymer for electrochemical actuators as it, so far, has outperformed other materials (Table S1, Supporting Information^5^), with reversible dimensional changes reaching 2% and 40% for the in‐plane and out‐of‐plane directions, respectively, when characterized from thin film configurations. Although the PPy backbone is hydrophobic, films that are manufactured via electro‐polymerization have a porous structure ensuring sufficient ion transport and water uptake. Recently, the engineering of hydrophilicity in conjugated polymers backbone has received significant attention as improved ion transport can enhance the performance in electrochemical devices for medical and energy storage applications.^6^, ^7^ In particular, ethylene glycol based side chains have been identified to enhance the transport of hydrated anions^7^, ^8^, ^9^ since those molecular moieties can strongly interact with water to promote swelling of the resulting polymer.^8^, ^10^ In these systems the initial hydration depends on the glycol chains content,^8^ morphology,^9^ and type of electrolyte^10^ while the swelling during electrochemical cycling has not been studied extensively.

Hydrogels on the other hand, can be engineered to exhibit reversible swelling upon chemical or physical stimuli.^11^ They typically consist of non‐conjugated polymer backbones with pending polar functional groups such as alcohols or carboxylic acids and, in some cases, enable volume expansion up to 1000–10 000%.^12^ The degree of swelling includes a shift of the equilibrium, which is dictated by the changes on water uptake, polymer conformation, solubility, and in crosslinking density.^13^ In the case of pH‐responsive hydrogels, pH is dictating the interaction between the polymer chains via hydrogen bonding and electrostatic interactions.^14^ While in the case of temperature responsive hydrogels, temperature impacts the solvation state of the hydrogel as a result of the interplay between intra‐ and inter‐molecular hydrogen bonding versus solubilization in water.^15^

Direct electric control over gelation that results in large, and potentially also reversible, volumetric changes has so far not been reported. Motivated by the idea to merge the concept of electrochemical actuation of conjugated polymer films with that of stimuli‐responsive gels, we aim to achieve electronic and reversible control over the solid‐to‐gel phase transition, and to achieve electronic control over large volumetric changes of a defined bulk. Such switching could be achieved if electric addressing controls the number of entanglement sites of the polymer and/or impacts its equilibrium with the solvent. In this work we report the electronic control over unprecedented volumetric switching of poly‐[3,3′‐bis(2‐(2‐(2‐methoxyethoxy)ethoxy)ethoxy)‐2,2′‐bithiophene], p(gT2), a conjugated polymer comprised of a polythiophene backbone with triethylene glycol side chains.^6^ p(gT2) changes its physical state from that of a conjugated polymer solid film to a gelled state as it is electrochemically doped, without the need of any chemical crosslinking or gelation processes.

Carbon monofilaments, of a diameter of 34.5 µm, were coaxially coated with p(gT2) (dissolved in chloroform) and were then redox‐switched in an aqueous KCl electrolyte solution. The p(gT2) forms a 5 µm thick solid cladding layer around the carbon fibers (**Figure** **1**). These cladding‐core fibers were then used as the working electrode with a Ag/AgCl wire as the counter electrode (Figure 1a,b). As processed, p(gT2) is in its neutral state. When a positive voltage is applied to the fiber, charges (holes) are accumulated along the conjugated polymer chains which then are compensated by chloride anions, from the electrolyte that enter into the film. Oxidation makes the p(gT2) cladding to expand while the reverse process forces the polymer to contract. The volumetric switching characteristics of p(gT2) were estimated by measuring the p(gT2)‐carbon fiber diameter, as recorded from micrographs, when p(gT2) was recurrently switched between its oxidized and neutral state. To simplify calculations, we extract the average p(gT2)‐carbon fiber diameter by assuming a homogenous cladding thickness and volumetric switching characteristics along the entire p(gT2)‐carbon fiber electrode (Figure S1, Supporting Information, and details in Experimental Section).

**Figure 1:**
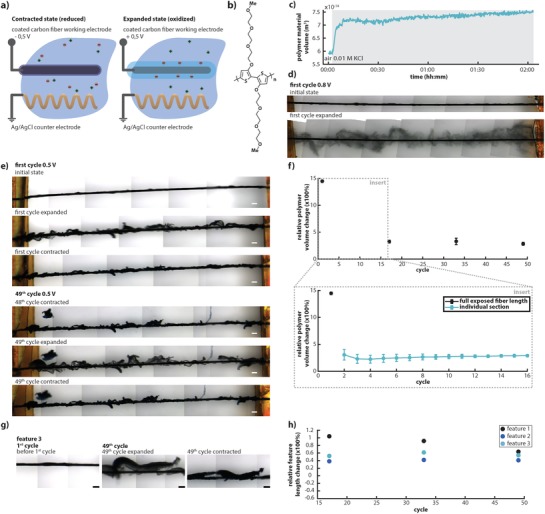
Large volume changes in p(gT2) during electrochemical switching. a) Electrochemical setup for characterizing the volume change of the polymer. b) Chemical structure of p(gT2). c) Passive swelling behavior of a p(gT2) coated fiber when immersed in electrolyte. d) p(gT2) coated fiber prior applying voltage and in oxidized state at +0.8 V. e) p(gT2) coated fiber in the initial unswitched state, at the expanded (+0.5 V) and contracted (−0.5 V) state during the 1st and 49th electrochemical switching cycle (scale bar 100 µm). f) Relative volume change of the polymer in the expanded state (+0.5 V) in respect to the previous contracted state (0.5 V) as a function of cycle number Δ*V_n_*/*V* ^pol^ *_n_*_−1_contr_ = (*V* ^pol^ *_n_*__exp_ – *V* ^pol^ *_n_*_−1_contr_)/*V* ^pol^ *_n_*_−1_contr_). Data in black correspond to the full length of the fiber (multiple frames) while data in blue are calculated from single frames covering a part of the fiber. g) Micrographs of specific features representing the polymer length change during electrochemical switching. h) Polymer length change during electrochemical switching.

When the polymer is oxidized for the first time (*n* = 1) at +0.8 V, the relative polymer volume change Δ*V*_1_/*V* ^pol^_0_contr_ reaches 12 000% (Figure 1d; Figures S2 and S3, Supporting Information) where Δ*V*_1_/*V* ^pol^_0_contr_ is defined as the polymer volume change (Δ*V*_1_ = (*V* ^pol^_1_exp_–*V* ^pol^_0_contr_) with respect to the initial unswitched state (*V* ^pol^_0_contr_). During this first switch a significant alteration in the p(gT2) morphology occurs, which appears to transform it from a solid‐state cladding layer into a gel phase (see Figure 1d). When the polymer is then subsequently reduced (at −0.8 V) it contracts, in a slightly non‐homogenous fashion. Parts of the cladding layer remain in its expanded state and appear as a diffused polymer network loosely confined around the central fiber, while other parts of the cladding seem to contract (Figure S2, Supporting Information). Repeated doping and de‐doping at ±0.8 V, versus the counter electrode, is at the limit of the polymer's electrochemical window (Figure S4, Supporting Information) and results in partial degradation of the polymer due to overoxidation and possibly also due to lower mechanical integrity.^6^ This very large but irreversible volumetric switching that occurs during its first oxidation process suggests that the ±0.8 V region can be explored in, for example, single‐switch electro‐valve applications.

Next, we investigated the switching properties and stability of the p(gT2) cladding within the ±0.5 V potential window, that is, within its safe electrochemical window (Figure S4, Supporting Information). When, the polymer is oxidized for the first time at +0.5 V, still a major initial relative volumetric expansion of Δ*V*_1_/*V* ^pol^_0_contr_ ≈ 1400% is observed (Figure 1e,f). For the subsequent red‐ox cycling (+/−0.5 V) (*n* ≥ 2 cycles) the relative polymer volume change Δ*V_n_*/*V* ^pol^ *_n_*_−1_contr_ = (*V* ^pol^ *_n_*__exp_–*V* ^pol^ *_n_*_−1_contr_)/*V* ^pol^ *_n_*_−1_contr_) of p(gT2) stabilizes (Figure 1e,f and Figure S5, Supporting Information). From the second cycle (*n* > 1) and on, typically, the polymer exhibits reversible Δ*V_n_*/*V* ^pol^ *_n_*_−1_contr_ values in the range from 280% to 330%. The stability of the volumetric changes was evaluated up to 300 cycles for redox cycling between +0.5 V and −0.2 V, (Figure S6, Supporting Information). Although the magnitude of the relative polymer volume change was slightly reduced due to a higher cycling frequency the material maintained its switching capacity over the 300 cycles.

From the micrographs (Figure 1e), we observe that the coating and/or its adhesion properties are in fact not perfectly homogenous along the carbon core fiber. Hence, the level of absolute expansion varies along the fiber extension (total length of ≈5 mm). However, we typically observe the same level of relative expansion‐contraction when comparing different positions along one fiber and in between different fibers. In order to directly compare the volumetric switching properties of p(gT2) with Polypyrrole we electropolymerized a 4.5 µm thick film on a carbon fiber and evaluated the volume changes with the same method used for p(gT2). Indeed, we observed a much smaller relative volume change of maximum 40% in the second cycle that is irreversible and a reversible change of 17% in later cycles. This is in agreement with values reported in literature (Table S1, Supporting Information).

Furthermore, in an effort to estimate also the change in length of the p(gT2) cladding layers, we decided to investigate specific features (one example is given in Figure 1g) that occasionally appear during the first oxidation (expansion) process. These features can reach a few hundred micrometers in length and the relative change in the length of these features upon redox‐switching reaches an average value of 61%, (Figure 1h). In addition, some of the features are barely attached and physically/electronically in contact with the carbon fiber but still demonstrate complete switching. This suggests that the polymer has sufficient electronic conductivity at low degrees of doping to support fast transport and accumulation of holes upon oxidation, thus the p(gT2) material promise for application as a free‐standing combined electrode‐actuator material for giant‐volume switching applications.

In order to understand the mechanisms behind the observed record high volume change values of the p(gT2) material and to confirm the hypothesized solid‐to‐gel (oxidation) and gel‐to‐solid (reduction) phase transitions, we examined the change in mass and viscoelasticity upon redox‐switching using QCM‐D. QCM‐D (Quartz Crystal Microbalance with dissipation monitoring)^16^, ^17^ is a common method used to monitor changes in the mass of a thin film due to the inclusion of ions and water and is also useful to monitor changes in the viscoelastic properties. QCM‐D has been applied to study both hydrogels^18^, ^19^ and conjugated polymers^20^, ^21^ in the past. Recently QCM‐D has been used to investigate swelling in conjugated polymers with glycol side chains^9^, ^22^ as well. A thin film of p(gT2) was coated on a QCM substrate with Au layer and mounted in the electrochemical cell of the QCM‐D. By monitoring the change in frequency (Δ*f*) and dissipation (Δ*D*) the evolution of the mass and the viscoelastic properties was recorded, respectively.^17^ As we suspect that the solid‐state film transforms into a gel phase there should be an obvious increase in viscoelasticity and mass, primarily dictated by the hydrodynamic coupling of water. By coating the Au quartz surfaces with p(gT2), the addition of the film generates a shift in frequency (1st overtone) of ≈300 Hz (see **Figure** **2**). Upon oxidation at +0.5 V, this Δ*f*_1st_ shifts an additional ≈700–1000 Hz at the same time as the overtones diverge, which indicates a considerable change in mass and also suggests a major increase in elasticity.^23^ The prime impact from oxidation on the p(gT2) film is also evident from a large change in Δ*D*. The shift in the dissipation value reaches 3–10 1E‐6 (overtone^−1^) (all overtones) and 50–250 1E‐6 (overtone^−1^) (1st to 9th overtone), respectively, upon adding the film and then by oxidizing it, respectively. Further, for the 11th and 13th overtone, we find that the signal is out of range, for both Δ*f* and Δ*D*, thus confirming that a very large change in volume and elasticity occurs upon oxidation. By then reducing the p(gT2) back to its neutral state, the initial thin film properties are to a large extent recovered (see Figure 2). Δ*D* returns almost entirely to its initial value, which indicates that the film returns to its solid phase but Δ*f* returns close to the initial value due to some remaining inclusion of electrolyte/water. In the second cycle, the same trend is once again observed (see Figure 2a). However, the mass and elasticity increase even further upon oxidation and the subsequent reduction switch generates a p(gT2) film that includes relatively more electrolyte/water. We choose to treat the QCM‐D data only qualitatively as the large swelling, induced during electrochemical oxidation, prevents us from fitting the data with good accuracy since QCM‐D provides valid information for films only within a tight dimensional window, of up to 250 nm.^16^

**Figure 2:**
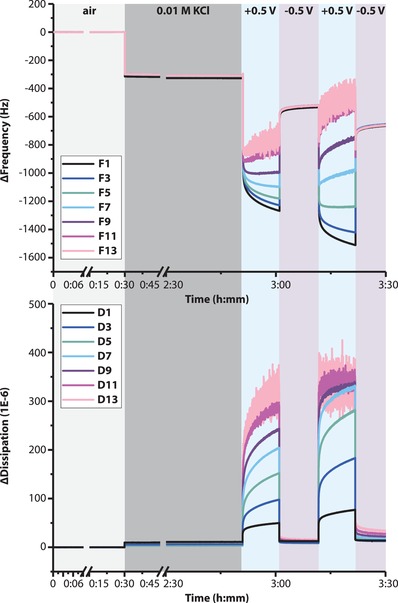
QCM‐D characterization of the polymer volume changes. Frequency and dissipation response to electrochemical switching in 0.01 m KCl (Δ*F_n_*/*n* and Δ*D_n_*/*n*).

To further understand the impact from redox‐switching on the phase transition of the p(gT2), molecular dynamics simulations were performed. **Figure** **3**d–g (first column) displays a system, comprising 250 p(gT2) chains (20 monomer units each) confined within a 23 × 23 × 18 nm^3^ box filled with water, where *n_i_* defines the number of positive charges per polymer chain. We choose *n_i_* = 6 to be the highest reversible oxidation state of p(gT2) based on density functional theory (DFT) calculations.^6^ As the polymer chains oxidize, they become more loosely packed and the film swells due to inclusion of ions, water and reorganization of the polymer chains, as illustrated by snapshots in Figure 3d–g (second column). At the neutral state, *n_i_* = 0, no swelling of the polymer film is observed, with basically no water molecules entering the film, but rather with water surrounding the side chains that are located along the outer surface. This agrees with the experimental observations where little swelling is observed when the film is immersed into an electrolyte prior to electrochemical switching (26% in volume, Figure 1c). As the oxidation level increases, water is penetrating the p(gT2) film, to finally reach the core of the volume. In the “fully” oxidized state, *n_i_* = 6, we observe the expansion of the volume to reach 410% based on the volume that ions and water molecules occupy inside the polymeric film, as calculated using a void‐versus‐polymer ratio approach (Figure 3d–g (last column)). However, the change in volume is relatively smaller (a factor of 3.4) as compared to our experimental results for the first switch at +0.5 V. This theoretical underestimation arises from the molecular dynamics (MD) simulations limitations imposed by the size of the box. Already at *n_i_* = 3 the polymer (Figure 3f) is reaching the boundaries of the box during expansion and at *n_i_* = 6 the polymer film is swelled to an extent, such that the polymer chains are distributed throughout the entire simulation box (Figure 3g). Therefore, we cannot fully monitor the expansion of the polymer in its full swelling magnitude.

**Figure 3:**
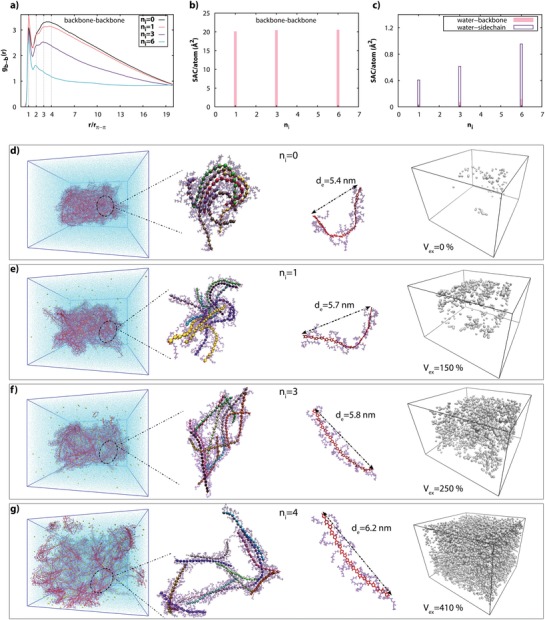
Molecular dynamics simulations of polymer swelling and water intake. a) Radial distribution of backbones *g*_b‐b_(*r*) as a function of the distance between the center of the mass of the backbones, where *r*_π–π_ = 0.39 nm is the π–π stacking distance between the chains. b,c) The surface area of a contact per atom in Å^2^ for b) backbones of the polymer, and c) water and backbones and water and side chains. d–f) Representation of the system from molecular dynamics calculation for different oxidized states *n*_i_ = d) 0, e) 1, f) 3, and g) 6; is a number of charges per chain. First and second column in d–g): Snapshot of the system where backbones are shown in red color, sidechains are shown in purple color, water molecules are shown in blue color, and ions are shown in yellow color. Third column in d–g): a representation of a polymer chain where *d*_e_ is the average end‐to‐end distance of the polymers at a given oxidation level. Last column in d–g): void volume inside the polymer matrix are shown in silver color which also represent the water molecules inside the polymer where *V*_ex_ represent the percentage of volume expansion.

Next, we study the conformational changes of the polymer chains and their mutual interactions. When the oxidation level increases, the polymer chains become more relaxed and straighter. This is quantified by the calculated average end‐to‐end distance of the polymers *d_e_*, which increases as the oxidation level increases, Figure 3d–g (third column). The volume expansion of the polymer can be traced in the backbone‐backbone distribution function *g*_b–b_(*r*) (Figure 3a). In the neutral state *n_i_* = 0 it shows a broad peak centered around *r*/*r*_π–π_ ≈ 4, with *r*_π–π_ = 0.39 nm being the π–π stacking distance. As the oxidation level increases this peak gradually diminishes, signifying that the distance between the chains increases. At all oxidation levels, *g*_b–b_(*r*) exhibits a sharp peak at *r*/*r*_π–π_ = 1 along with much weaker peaks at *r*/*r*_π–π_ = 2, 3 (note that the latter peaks are superimposed on a broad background peak centred at *r*/*r*_π–π_ ≈ 4 as discussed above). These sharp peaks reflect a formation of small crystallites mostly composed of two π–π stacked chains (corresponding to *r*/*r*_π–π_ = 1), which can be clearly seen in snapshots in Figure 3d–g (third column). Figure 3b shows a calculated surface area of a contact per atom for backbone of the polymer chains. It does not change as n_i_ varies, which provides a clear evidence that the chains stay effectively interconnected for all oxidation levels. Note that we also calculated the distribution functions and contact areas for sidechains and ions (Figure S9, Supporting Information), and they exhibit a similar behavior as those shown in Figure 3a–c. Hence, the MD simulations show that the change in volume is due to an extension of the polymer chains and an increase of a distance between them, keeping the chains interconnected, allowing the side chains to solvate and to include a relatively larger amount of water and ions.

The fact that the polymers maintain π‐π stacked in the gelled state justifies that the material is still conducting as percolation pathways are preserved for the electronic carriers. Using multiscale transport calculations it has been demonstrated recently that an effective π‐π stacking between the chains giving rise to a percolative network over the whole sample is a prerequisite for a high mobility in conducting polymers.^24^

Let us now discuss the microscopic origin of the swelling behavior. We argue that this process is mainly governed by electrostatic interaction between charged polymer and counterions and related to the water intake facilitated by the counterions entering the polymer film during oxidation. As described in the Experimental Section, in the MD simulations we mimic the experimental oxidation by adding positive charges to the polymer backbone, simultaneously adding negative counterions into water outside polymer in order to maintain the overall charge neutrality. In order to outline the role of counterions and electrostatic interaction in swelling, we performed the following numerical experiment, artificially changing the condition for the swelling and water intake. Namely, for each given oxidation level, instead of placing the counterions into water, we added them inside the polymer and let the polymeric film to equilibrate. After that we placed the resulted polymeric film into the water and monitored swelling and water intake. In this case, for all oxidation levels studied, the polymer film did not show any swelling (see Figure S10, Supporting Information). The difference between these two models is that in the first model (Figure 3), the polymer films become positively charged during the oxidation. Hence, negative chloride anions are forced by electrostatic forces from the surrounding water into the film to compensate the positive charges. The anions are surrounded by hydration shells while diffusing in a polymer.^25^ Hence, the negative counterions bring along water molecules into the polymer film making it to swell. On the contrary, swelling does not take place for the second model (Figure S10, Supporting Information), because the polymer film remains electrically neutral and there are no counterions outside the film that can bring water inside it. Note that this behavior is fully consistent with the results for the first model at zero oxidation level, where no swelling is observed for the case of fully reduced polymer (i.e., when a neutral polymer is immersed in water, see Figure 3d).

It is also noteworthy that hydrophobicity and hydrophilicity of different parts of polymers are also important for swelling. The backbone of the polymer is hydrophobic whereas the sidechains are hydrophilic (this is illustrated in Figure 3c showing the calculated surface area of contact per atom for water and backbone‐water and sidechains‐water). On one hand, water can easily penetrate the polymer because of the hydrophilicity of the sidechains. At the same time the hydrophobic backbones avoid water and hence help to form gel‐like structure by keeping the connections between them and preventing the material from getting dispersed in water. Using the MD simulations we also studied a reverse process of de‐swelling when the polymer is reduced back to its neutral state in the second part of the voltammetry cycle. In a full agreement with the experiment we find that the volume of the polymer shrinks; however, its final volume remains larger than the initial one, with the volume expansion V_ex_ ≈ 80% (see Figure S11, Supporting Information). We attribute the persistence of swelling of the neutral state at the end of the first cycle to the presence of the water inside the polymer, which cannot be completely removed from the film in the course of the voltammetry cycle.

The large magnitude of volumetric switching upon electrochemical addressing, here reported, is perhaps best utilized in aqueous or electrolyte‐based device applications where electro‐actuation of large displacements or change in volume are desired. As a proof of concept, we constructed an electro‐active filter or sponge device, of which the size of the pores can be controlled electrically (**Figure** **4**). The surface of a carbon sponge, with the average pore size of 250 µm, was coated with a cladding of the p(gT2) material. During oxidation (+0.5 V) the polymer expands and the pores of the resulting filter sponge become smaller, thus reducing the initial porosity of the sponge by 85%, as recorded after the 3rd switching cycle (Figure S12, Supporting Information). Depending on the size of the pore and the thickness of the initial coating the pores either close completely or partially (see Figure 4). Further, the porosity can be reversibly modulated in subsequent cycles by about 60% (Figure S13, Supporting Information). In this simple demonstration, we show a potential practical implication of the p(gT2) polymer. So, for the first time ever, we report the electronic control over the porosity via electrochemical doping of an electro‐active material. As the polymer supports sufficient intrinsic conductivity, the polymer can potentially also be applied to non‐conductive substrates.

**Figure 4:**
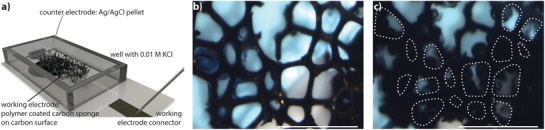
Electrochemical change of pore size in a p(gT2) coated carbon sponge. a) Experimental setup for pore size variation experiments. b) P(gT2) coated carbon sponge before electrochemical switching. c) P(g2T) coated carbon sponge in oxidized state, *V* = +0.5 V (3rd cycle) (circles indicate the initial pore size) (scale 1000 µm).

In conclusion, we report a conjugated polymer, p(gT2), including ethylene glycol side chains, that can be transformed in between a solid state and a gelled state, upon oxidation‐reduction switching. When shaped as a cladding layer around a carbon fiber, the initial application of +0.5 and +0.8 V, versus Ag/AgCl electrode, makes p(gT2) to gel and swell 14 and 120 times, respectively, with respect to its pristine thin film state. For consecutive switching at ±0.5 V the material typically exhibits a reversible swelling of 300%, when referring to its previous contracted state. The electronic control over volume of p(gT2), here presented, outperforms prior reports on conjugated polymer systems commonly explored for actuators, such as PPy, and we identify several interesting future pathways based on electronic solid‐to‐gel switching materials and devices. Upon application of oxidation‐reduction potentials, the system is brought out of equilibrium thus promoting transition to occur between the solid state and the gel phase. Using molecular dynamics simulation, the microscopic origin of the swelling behavior was unraveled. We argue that this process is mainly governed by electrostatic interaction between charged polymer and counterions and related to the water intake facilitated by the counterions entering the polymer film during oxidation. With the inclusion of dedicated cross‐linking sites, composite components and/or co‐polymer units, we foresee strategies to optimize the fundamental volume‐switching characteristics, in the aspect of stability, switch speed, and volumetric range. Further, by improving and advancing device architectures, based on “*e*‐swelling” materials, for instance including strain relaxation, improved conductivity, or the guidance of swelling to occur in one particular direction, one can imagine several novel actuator devices for fluidic control, release of materials, filtering, or even gel robotics. Here, we report one novel approach of a smart sponge, of which we can electronically tune the porosity by 60–85%.

## Experimental Section

*Synthesis of p(gT2)*: Polymer p(gT2) was synthesized as described in the literature.^6^

*Coating of Carbon Materials with p(gT2)*: Carbon fibers (Carbon monofilament, Specialty Materials, Lowell, MA, USA) were treated by UV ozone for 15 min (PSD‐UV, Novascan). The pretreated fibers were mounted to the shaft of a small motor, while the other end of the fiber was inserted into a glass capillary. After the polymer was solubilized with chloroform, the polymer was fed into the capillary. While being rotated by the motor, the carbon fibers were then pulled out of the glass capillary containing the polymer. When the carbon fiber was pulled out of the capillary, the polymer was deposited on the fiber. The carbon fiber was turned by an electric motor to achieve a more uniform coating around the fiber. The coating procedure was performed under an Olympus SZ60 stereomicroscope for visual control. In case of the carbon sponge, the polymer was drop casted. The coated carbon materials were left to dry overnight before being used for experiments.

*Electrochemical Switching of p(gT2) Coated Fiber/Sponge with Simultaneous Monitoring under an Optical Microscope*: The fibers were mounted on a glass slide with Kapton tape. The coated sponges were attached to DropSens DS100 carbon electrodes through carbon paste and a PDMS well was installed around it to contain the electrolyte. The polymer was electrochemically addressed in a two‐electrode setup with an Ag/AgCl pseudo reference—counter electrode by a Metrohm µAutolab Type III (NOVA 2.1 software) (Figures 1a, 4a). Electrochemical experiments were performed in 0.01M KCl. The electrochemically induced polymer volume change was observed with a Nikon SMZ1500 stereomicroscope and a Nikon WD 45 lens and a Fiberoptic Heim LQ1600 light source in combination with a Nikon DS‐Fi1 camera and Nikon NIS Elements 3.22.15 software.

*Image Processing*: Images of the full active fiber length were assembled with Adobe Photoshop, the background removed, and the active part of the fiber extracted. The images were then loaded in MATLAB, transformed to grayscale, and the pixels below a grayscale threshold counted. The number of pixels below the threshold allow computing the average fiber diameter of the fibers. Assuming a cylindrical shape the volume of the fibers was calculated. Individual frames were processed in MATLAB based on their grayscale values as well. Since the background variation within one frame was usually much smaller than for assembled images of the full fiber length, it was usually possible to remove the images without prior manual removal of the background.

The total average diameter for the contracted and the subsequent expanded fiber, of a specific reduced or oxidized state, was estimated, respectively. The resulting total volumes, assuming rotational symmetry, were then calculated. After subtracting the carbon core volume, the polymer volume was calculated in its contracted (*V* ^pol^ *_n_*_−1_contr_) and expanded (*V* ^pol^ *_n_*__exp_) states for a given (*n*) red‐ox cycle. The resulting Δ*V_n_*/*V_n_*_−1_ = (*V* ^pol^ *_n_*__exp_ – *V* ^pol^ *_n_*_−1_contr_)/*V* ^pol^ *_n_*_−1_contr_) are then presented for each *n* ^th^ cycle, where *n* = 1 denotes the first cycle, switching from the pristine (unswitched) to the initial oxidized state and *n* > 1 denotes the following switch cycles. Details on the extraction of diameter and volume data can be found in Figure S1, Supporting Information.

The pore sizes of the sponge experiments were calculated from gray value thresholds as well. Lengths of specific features were measured with ImageJ.

*QCM‐D Characterization*: To determine the swelling of the polymer material both passive and upon electrochemical stimulation. Quartz crystal microbalance with dissipation (QCM‐D) experiments were performed (Q‐Sense 401 QCM‐D, Biolin Scientific). Initially the reference resonances of fresh Au quartzes (5 MHz fundamental frequency, Q‐Sense 301) in 0.01 m KCl was identified. These quartzes were then spincoated with the polymer (5 mg mL^−1^ in chloroform) at 3000 rpm. After being dried overnight, the actual QCM‐D experiments were performed. Electrochemical stimulation of the material was hereby performed in a three‐electrode setup with Ag/AgCl reference electrode and the counter electrode integrated in the QCM‐D chamber. Electrochemical stimulation was performed with a Metrohm µAutolab Type III.

*Molecular Dynamics*: All‐atom molecular dynamics (MD) simulations were performed to study the swelling behavior of the polymeric material in the presence of water and ions. All the MD calculations in the LAMMPS package^26^ were carried out and all the system using the moltemplate code was generated.^27^ Both bonded and nonbonded interactions of the atoms were described by general AMBER force field (GAFF) where TIP3P (transferable intermolecular potential 3P) are used to model the water molecules. Note that the GAFF was successfully used for description of many morphological features experimentally observed in π‐conjugated conducting polymers including the π–π stacking among the polymer chains, a crystallite size, a lamellar structure formation, effect of the substrate, and many others.^28^, ^29^, ^30^ Bonds and angles of water are constrained by SHAKE algorithm.^31^ The chain length of 20 repeating monomer units was taken where the triethylene glycol sidechains were connected to the alternate thiophene ring. Implicit solvent model was used to speed up the equilibration process as it can accelerate the conformational changes of the polymers.^32^ In order to mimic the dissolution of the polymers in the solvent chloroform, damping parameter of 3800 fs and dielectric constant of 5 were used.^33^ The electrostatic potential (ESP)^34^ derived partial charges of the polymers were calculated from density functional theory (DFT) calculations with ωB97XD^35^ functional and with 6–31+g(d)^36^ basis set as implemented in Gaussian.^37^ The force‐field was not modified for different charged states of the system.

a) *Generating Self‐Aggregated Polymeric Film*:

Initially 250 charge‐neutral polymer chains were randomly placed in a rectangular box of the size 80 × 80 × 60 nm^3^ without any position overlap where the box size was then gradually decreased 14 × 14 × 10 nm^3^ to have an approximately corresponding polymer density. Then the polymer chains were relaxed by an energy minimization step using conjugate gradient (CG) algorithm. The system was equilibrated for 5 ns in the isothermal‐isobaric ensemble (NPT) at a temperature of 503 K and at a pressure of 1 atm with the implicit solvent model of Langevin dynamics. The polymeric film of 250 neutral chains was made by performing simulated annealing with the effect of implicit solvent to replicate the self‐assembly of the polymers at room temperature. The equilibrated melt polymers were quenched from 503 to 303 K at a cooling rate of 10 K/200 ps. The system was then equilibrated at 303 K and 1 atm for 10 ns using an implicit solvent following ref. 33. The final structures were obtained without the effect of solvent by mimicking the dry‐phase. All the simulations were run at a timestep of 2 fs. (Note that the implicit solvent was used only to generate a film in order to accelerate the conformational changes; all calculation of swelling and water intake were done with the explicit solvent (water) as described below).

b) *Swelling of the Polymers*:

To study the swelling behavior, the generated charge‐neutral polymeric film was dipped into water. The equilibrated polymers were placed in the center of the simulations box of the size 30 × 30 × 24 nm^3^ which were surrounded by the water molecules. The system containing polymers and water was then equilibrated for 20 ns in the NPT ensemble at 303 K and 1 atm. The equilibration time of the simulation runs was chosen to ensure that the total and the potential energies reach a saturation. The final size of the simulation box was 23 × 23 × 18 nm^3^. The neutral polymeric film was oxidized by increasing the charges of each polymer chain from 0 to 6. To compensate the positive charges added in the chains and to make the total charge of the whole system equal to zero, exact number of chloride (Cl^−^) ions was added in the water outside the p(gT2) film. A reverse study of the de‐swelling was performed by starting with the fully oxidized structure *n_i_* = 6 and reducing (discharging) the polymer chains from *n_i_* = 6 to *n_i_* = 0. The simulation in the NPT ensemble was run at 300 K and 1 atm until the equilibrium was reached. To compensate the negative charges of Cl^−^ ions, the same number of Na^+^ ions was added in water outside of the film.

## Conflict of Interest

E.S., J.G., and M.B. filed a patent application related to this work in the EPO, EP16178248.7.

## Supporting information

Supplementary
