# Supplementary material for: Reversible Electronic Solid–Gel Switching of a Conjugated Polymer
Source: Adv Sci (Weinh). 2019 Oct 28;7(2):1901144. doi: 10.1002/advs.201901144 (PMC6974956; doi:10.1002/advs.201901144)
Supplement: Supplementary file 1 — Supplementary [file ADVS-7-1901144-s001.pdf]

Copyright WILEY-VCH Verlag GmbH & Co. KGaA, 69469 Weinheim, Germany, 2019.

Supporting Information

for *Adv. Sci.,* DOI: 10.1002/advs.201901144

Reversible Electronic Solid–Gel Switching of a Conjugated Polymer

Johannes Gladisch, Eleni Stavrinidou, Sarbani Ghosh, Alexander Giovannitti, Maximilian Moser, Igor Zozoulenko, Iain McCulloch, and Magnus Berggren**

Supporting Information

Reversible Electronic Solid-Gel Switching of a Conjugated Polymer

Johannes Gladisch[†], Eleni Stavrinidou[†], Sarbani Ghosh, Alexander Giovannitti, Maximilian Moser, Igor Zozoulenko, Iain McCulloch, Magnus Berggren[*]*

Table S1: Deformation in conjugated polymers upon electrochemical doping

Polymer	Dopant	Electrolyte Solvent	Testing device	Direction of deformation	Primary information		Dimensions
					1st cycle	consecutive	
**Polythiophenes**							
**Poly(quarterthiophene)**	PF_6, S-PHE [1]	TEA PF_6 in Acetonitrile	bilayer actuator	in plane	17.7-21.4%		80 µm thick bilayer actuator with total length of 2,25 mm where 0,15 mm bend
**Polypyrrole**							
**Polypyrrole (out of plane)**	DBS [2]	Na DBS aqueous	thin film	out of plane (thickness)		51% 1 µm<br>37% 5 µm<br>28% 10 µm	1 µm film<br>5 µm film<br>10 µm film<br>On d=500 µm gold cylinder<br>10 mm active surface
	DBS [3]	Na DBS aqueous	Thin film	Out of plane (thickness)	60-100%	30-40%	10 -20 $µm^2$ (e.g. 30 µm wide)<br>1-1.5 µm thick
**Polypyrrole (in plane)**	DBS [4]	Na DBS aqueous	thin film	in plane		2%	1.91 mm x 40 µm x 1 µm thick
**Polypyrrole/PEO mix**	DBS, PEO [5]	Li TFSI aqueous or PC	free standing films	along film		≤11.4%	15x1 mm<br>44±2 µm thick<br><br>1 mm active area between the clamps that drive the actuation measurement device

DBS = dodecyl benzene sulfonate
PC= propylene carbonate
PEO= Polyethylene oxide
S-PHE = Sulfated Poly(β-HydroxyEther)
TEA = Tetraethylammonium
TFSI = bis(trifluoromethanesulfonyl)imide

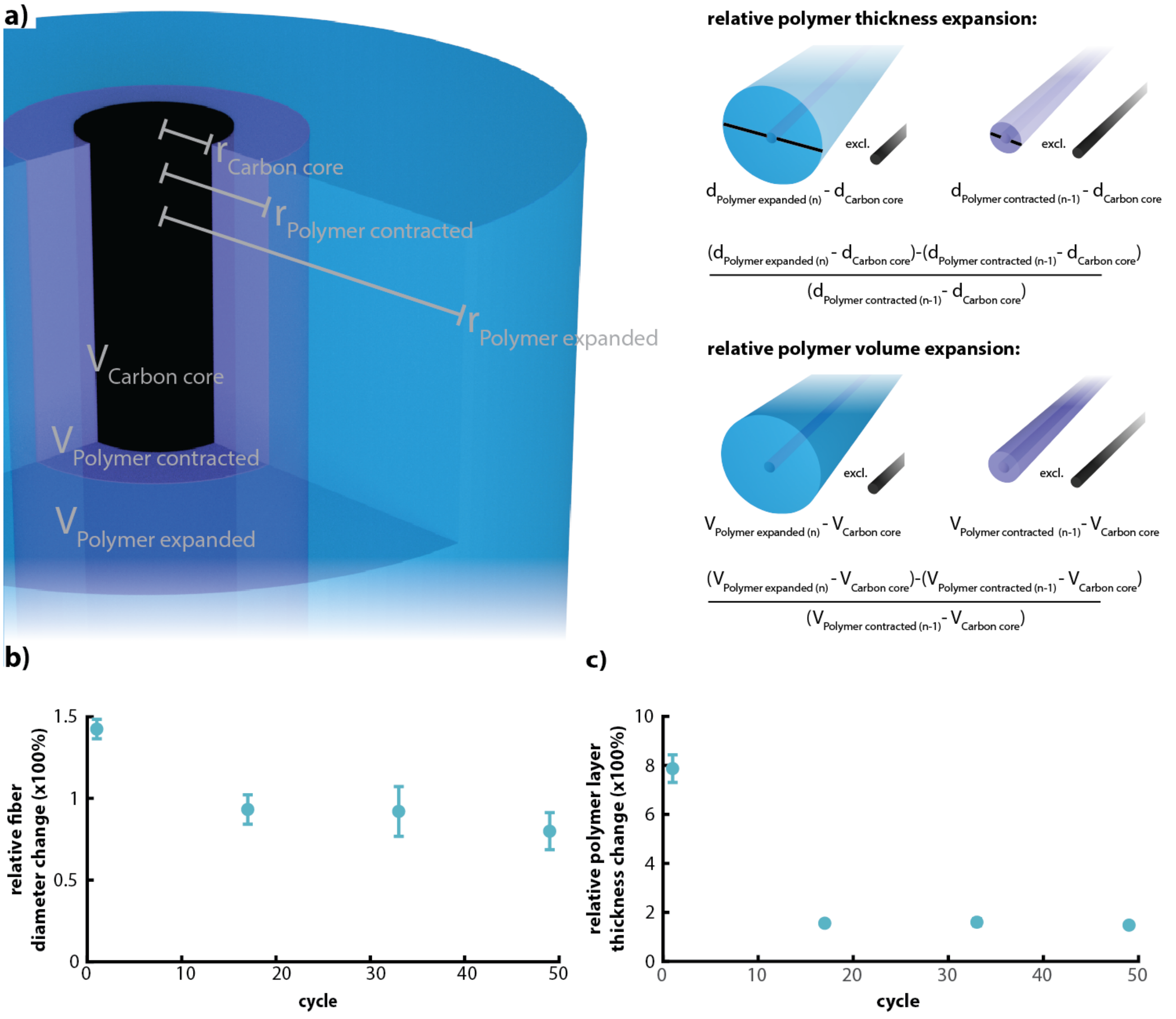

Figure S1: a) Structures used to calculate diameters and volumes of the relative expansion and contraction of the polymer. b) Relative total fiber diameter change when electrochemically switched at ±0.5 V. c) Relative polymer layer thickness change when electrochemically switched at ±0.5 V.

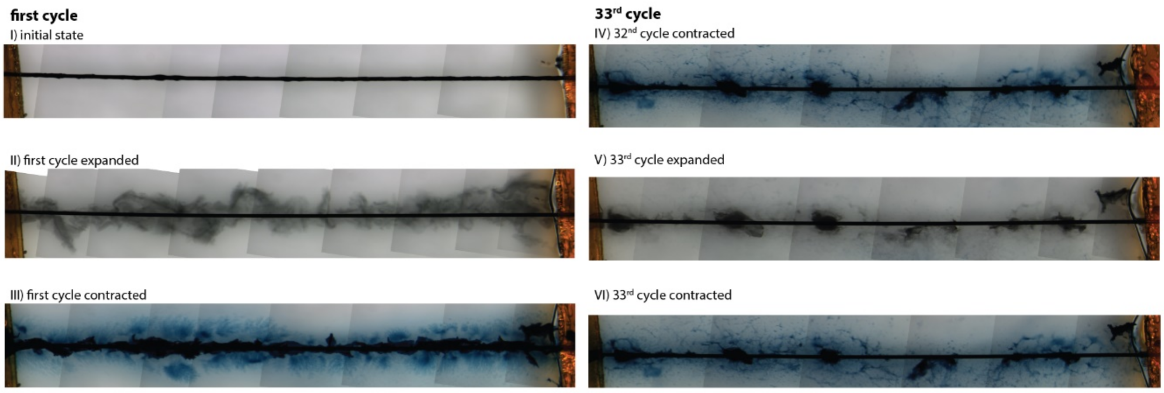

Figure S2: p(gT2) coated fiber in left: I. when immersed in the electrolyte with no voltage applied, II. in oxidized state at +0.8 V (as displayed in figure1) and III. in reduced state at -0.8

V right: the 33rd switching cycle contracted before (IV), expanded (V) and contracted afterwards (VI), respectively.

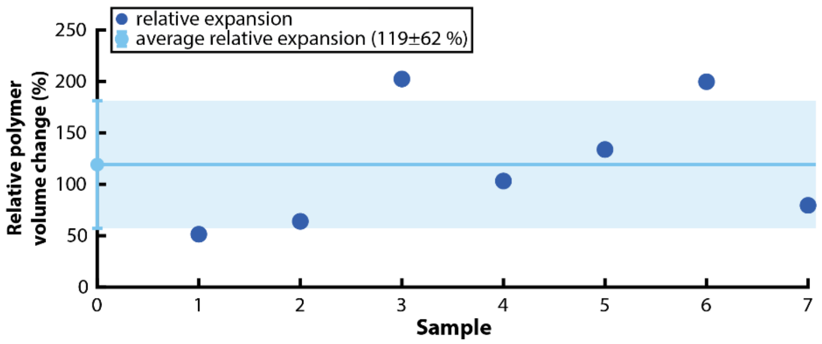

Figure S3: Relative polymer volume expansions of different fibers in the first cycle when +0.8 V is applied.

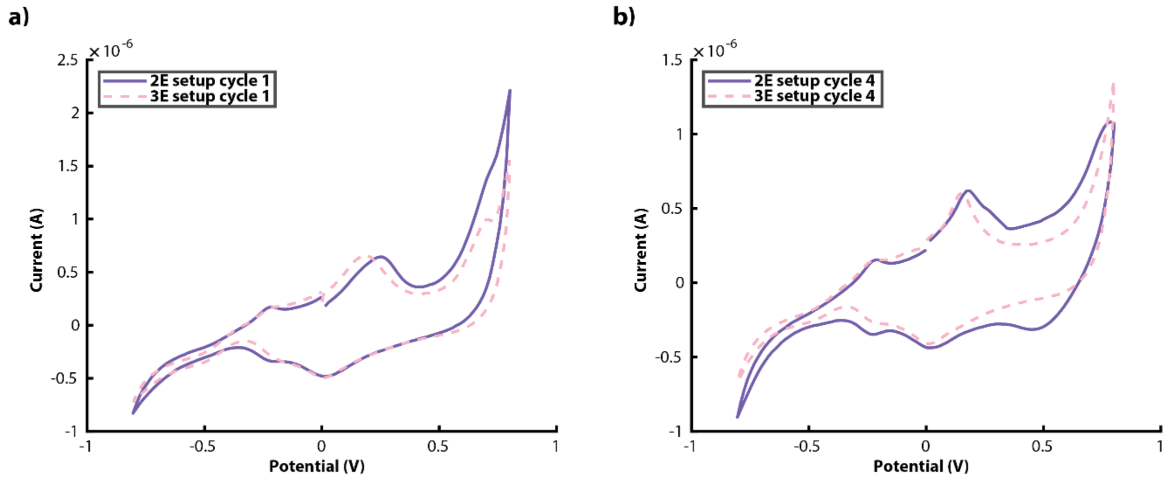

Figure S4: Cyclic Voltammograms of p(gT2) between -0.8 V and 0.8 V at 10 mV/s acquired with a 2-electrode setup (2E) and 3-electrode setup (3E).

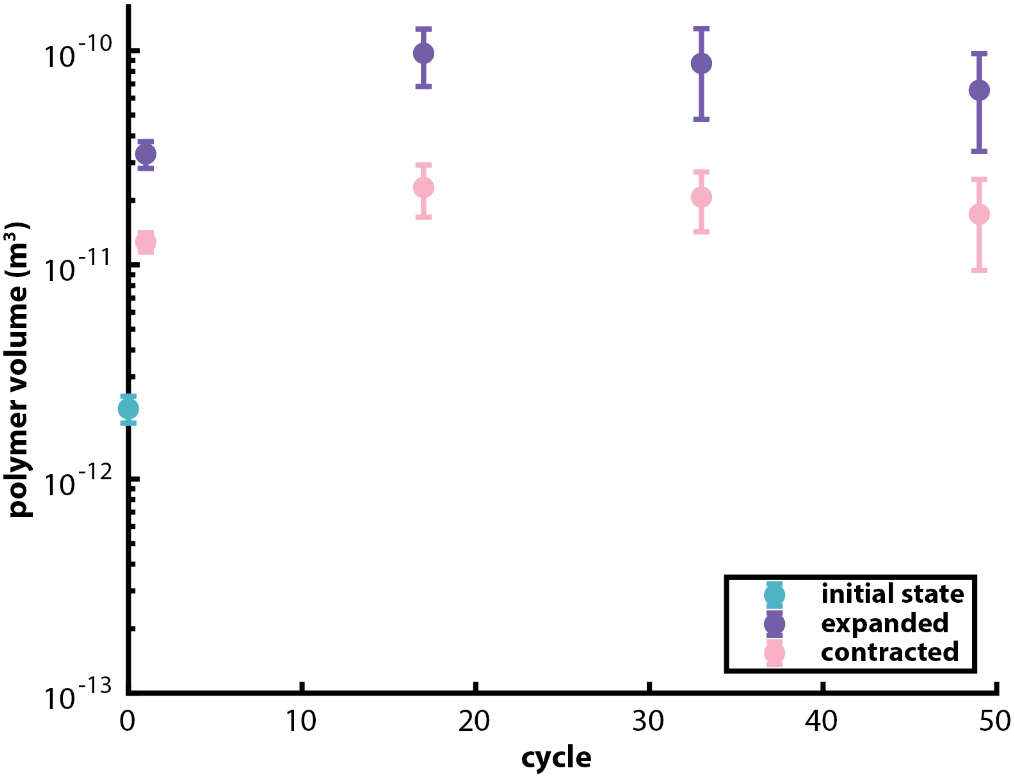

Figure S5: Total polymer volume as a function of cycle number. Initial state corresponds to the fiber before application of bias. At each cycle the total polymer volume in the expanded and contracted state is given.

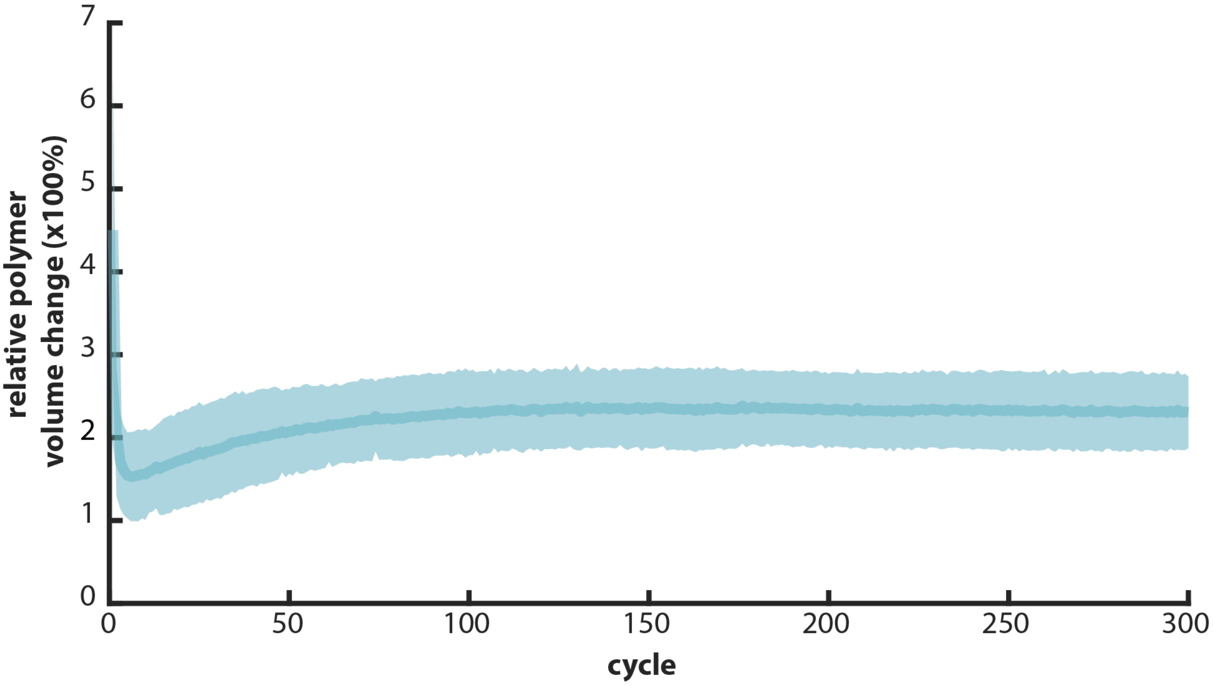

Figure S6: Relative volume change of the polymer in the expanded state (+0.5 V) in respect to the previous contracted state (-0.2 V) as a function of the cycle number, $\Delta V_n/V^{pol}_{n-1\_contr} = (V^{pol}_{n\_exp} - V^{pol}_{n-1\_contr})/ V^{pol}_{n-1\_contr})$. The addressing protocol for one cycle is +0.5 V for 30 sec

followed by -0.2 V for 30 sec. The experiment was performed with a three-electrode setup with a Pt counter electrode and Ag/AgCl pseudo reference electrode in 0.01 M KCl electrolyte.

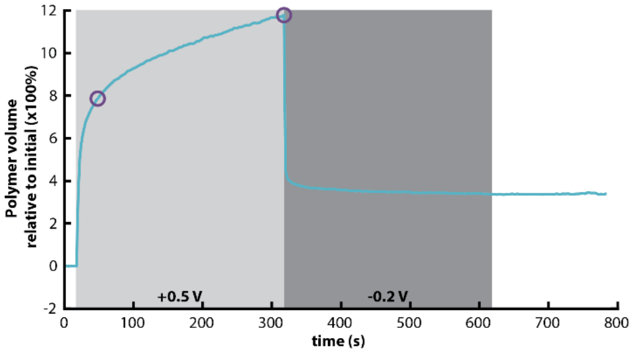

Figure S7: Temporal evolution of the relative volume change of P(gT2) in respect to the initial state (before biasing) during oxidation (+0.5 V) and reduction -0.2 V. Circles indicate values at 30 seconds and 5 minutes.

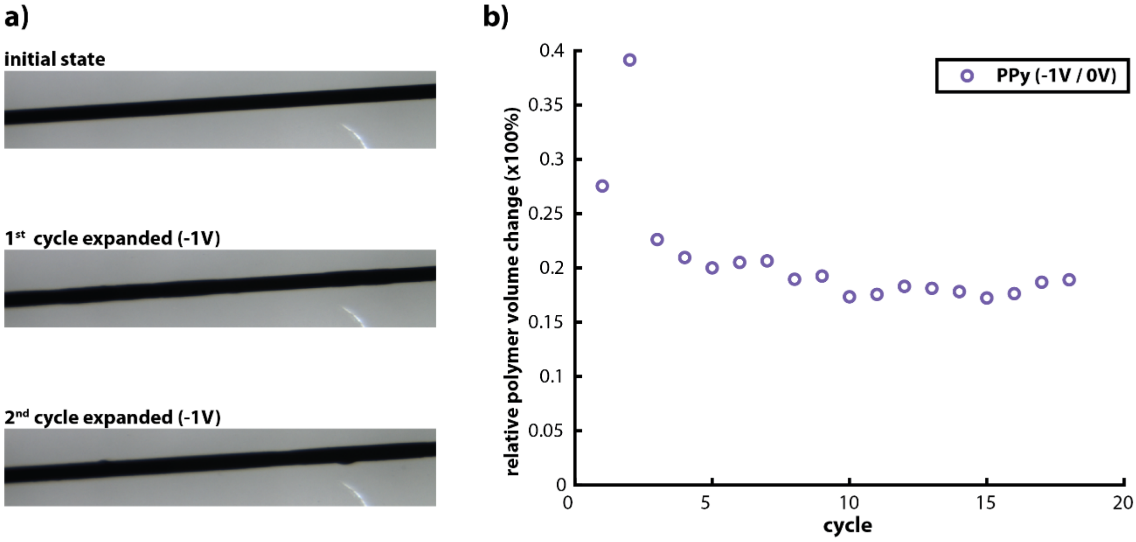

Figure S8: a) Ppy:DBS coated fiber in the initial unswitched state and in the expanded state (-1 V) during the 1st and 2nd electrochemical switching cycle in 0.1 M NaDBS. b) Relative volume change of the PPy in the expanded state (-1 V) in respect to the previous contracted state (0 V) as a function of cycle number $\Delta V_n/V^{pol}_{n\text{-}1\_contr} = (V^{pol}_{n\_exp} - V^{pol}_{n\text{-}1\_contr})/ V^{pol}_{n\text{-}1\_contr}$) (Cycle time 200 sec following [2]).

Polypyrrole was electropolymerized in a 3-electrode setup at 0.55 V from an aqueous solution of 0.1 M Pyrrole and 0.1 M NaDBS following a common recipe [6]. After electropolymerization for 10 minutes a film with comparable thickness to the p(gT2) coated fibers was obtained.

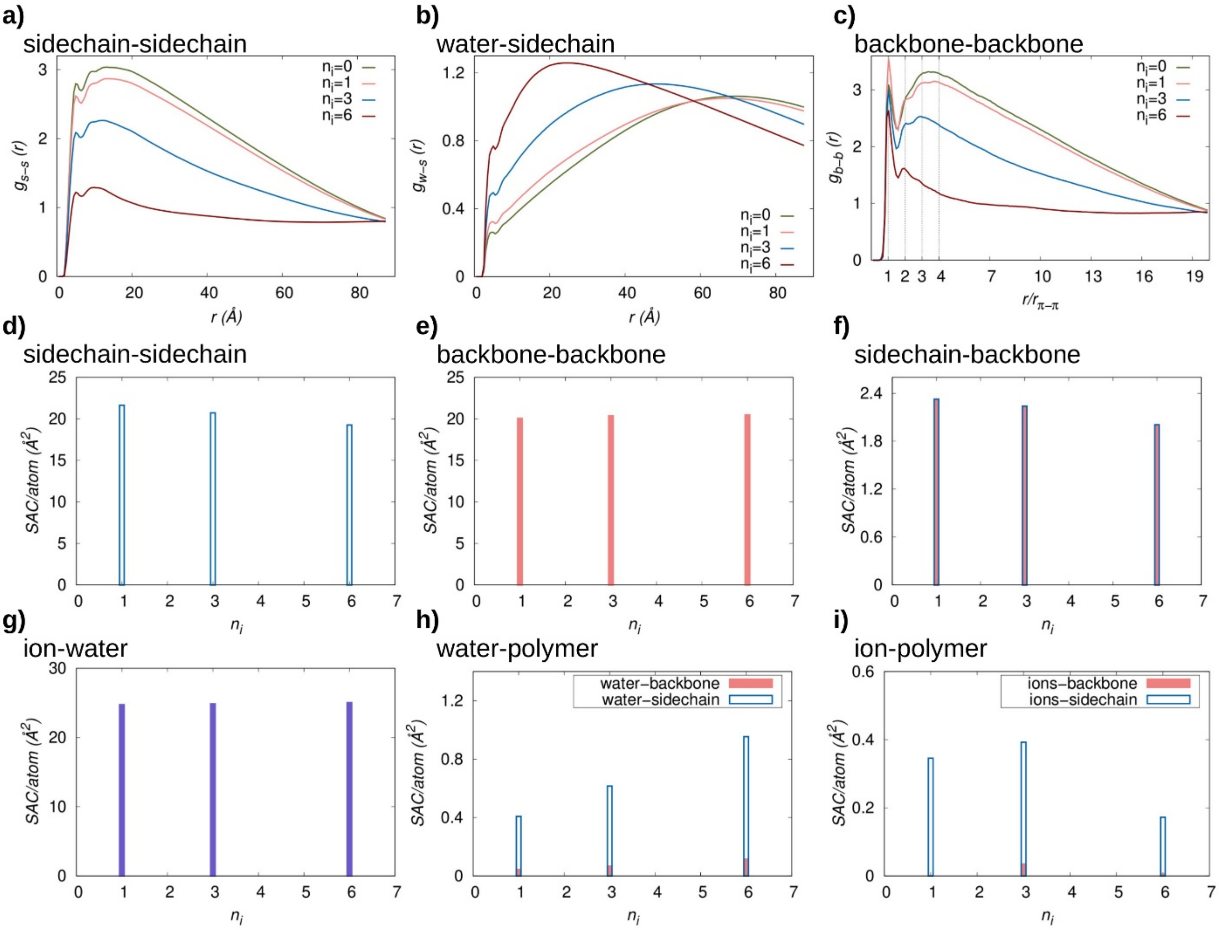

Figure S9: a) Radial distribution functions (g(r)) of sidechains as a function of distance (r), b) radial distribution of sidechains from water molecules as a function of r, c) radial distribution of backbones as a function of r where $r_{\pi\text{-}\pi}$ = 0.39 nm defines the π-π stacking distance of the backbone. Surface area of contact per atom in Å^2, a) among the sidechains of the polymers, b) among the backbones of the polymer, c) between the sidechains and backbones, d) between the water and ions, e) between water and polymer and f) between ions and polymer.

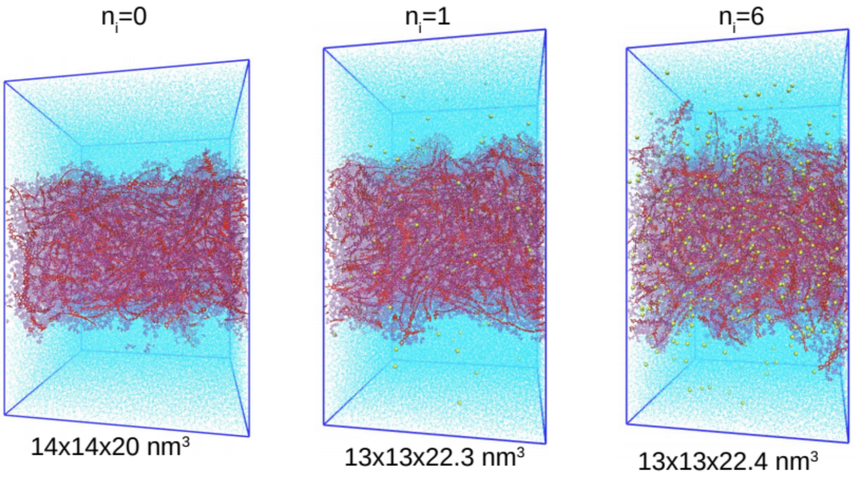

Figure S10: Snapshots of the MD simulations at different oxidation levels a) n_i= 0, b) n_i=1, and c) ni= 6. Note that in the present calculations the Cl^- counterions were placed first in the polymer, and then the equilibrated polymer film was immersed in water (see text for details).

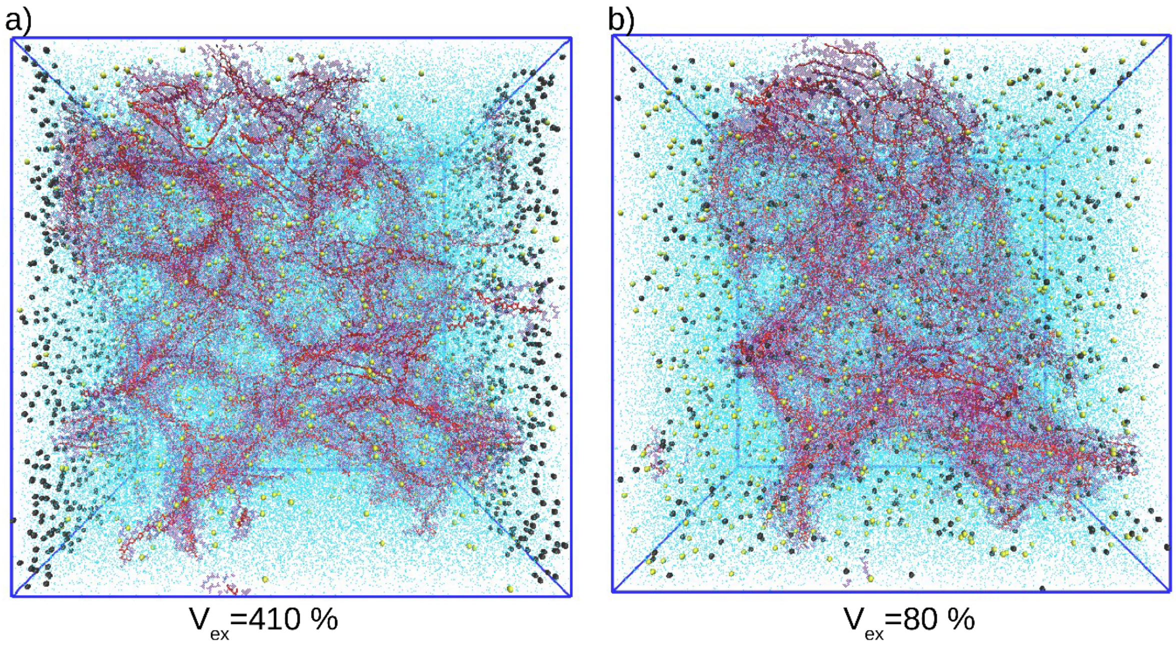

Figure S11: MD simulations of the de-swelling when the polymer is reduced back to its neutral state in the second part of the voltammetry cycle. (a) n_i=6 (fully oxidized state); (b) n_i=0 (fully reduced state). V_{ex} represent the percentage of volume expansion in comparison to the initial dry film.

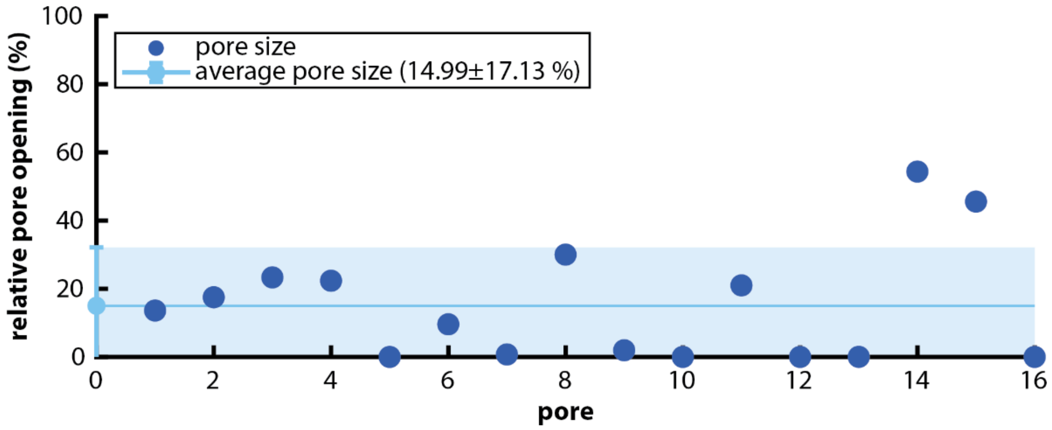

Figure S12: Relative pore opening in respect to the initial state (before application of voltage) of different pores of the coated sponge for cycle 3 at the oxidized state of p(gT2). 100% denotes the pore is fully open as in the initial state and 0% denoted the pore is fully closed.

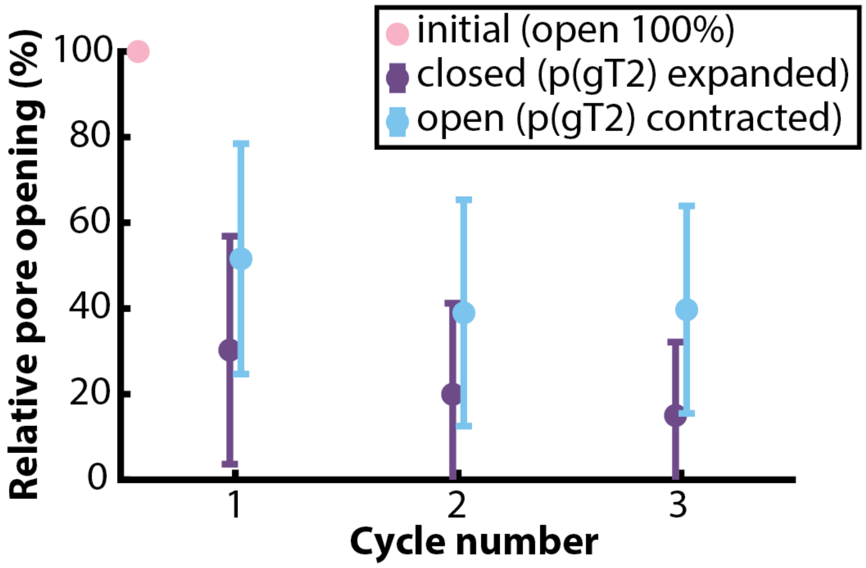

Figure S13: Average pore opening in respect to the initial state (before application of voltage) of the coated sponge during electrochemical switching of the p(gT2).

References:

[1] P. A. Anquetil, H. Yu, J. D. Madden, T. M. Swager, I. W. Hunter, *Proc. SPIE* **2003**, *5051*, 42.
[2] D. Melling, S. Wilson, E. W. H. Jager, *Smart Mater. Struct.* **2013**, *22*, 104021.
[3] E. Smela, N. Gadegaard, *Adv. Mater.* **1999**, *11*, 953.
[4] E. Smela, M. Kallenbach, J. Holdenried, *J. Microelectromechanical Syst.* **1999**, *8*, 373.
[5] R. Khadka, Z. Zondaka, A. Kesküla, M. S. Khorram, T. T. Khanh, T. Tamm, J. Travas-Sejdic, R. Kiefer, *J. Appl. Polym. Sci.* **2018**, *135*, 46831.
[6] E. Smela, N. Gadegaard, *J. Phys. Chem. B* **2001**, *105*, 9395.
